# Supplementary material for: Development of microglia-targeting adeno-associated viral vectors as tools to study microglial behavior in vivo
Source: Commun Biol. 2022 Nov 11;5:1224. doi: 10.1038/s42003-022-04200-3 (PMC9652230; doi:10.1038/s42003-022-04200-3)
Supplement: Supplementary file 2 — Supplementary Information [file 42003_2022_4200_MOESM2_ESM.pdf]

Supplementary Figure 1

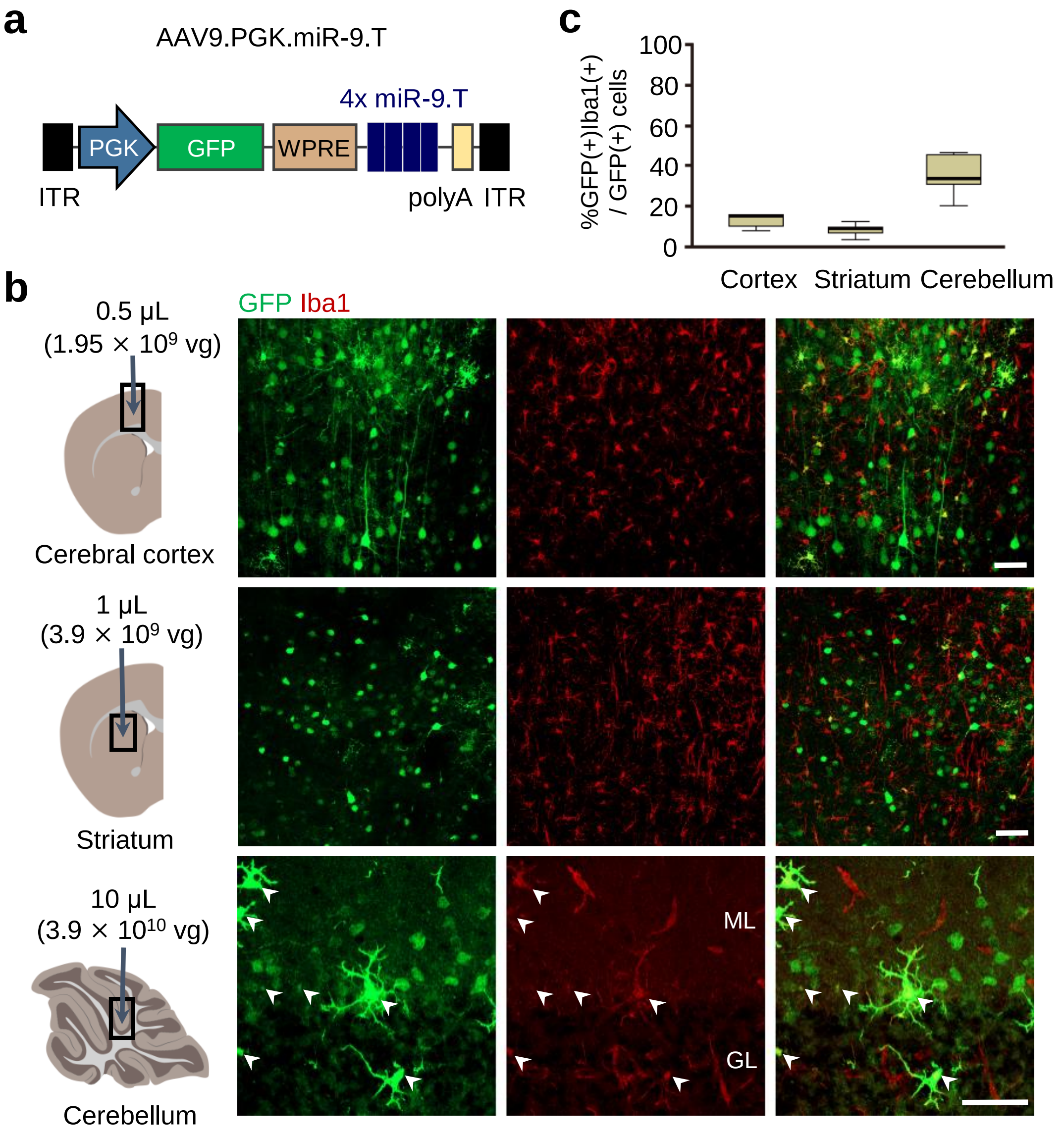

Supplementary figure 1. Numerous non-microglial transduction events by AAV9.PGK.miR-9.T. (a) Schema of AAV genome, which was designed to express GFP and four juxtaposed miR-9 target sequences (miR-9.T) under the control of the PGK promoter. **(b)** Adult mice received an injection of the AAV vectors to the cerebral cortex, striatum, and cerebellum as illustrated. Right panels show immunohistochemistry of slices of the cerebral cortex (upper), striatum (middle), and cerebellum (lower) for GFP (green) and Iba1 (red) one week after the viral injection. Arrowheads in lower panels indicate cells labeled with both GFP and Iba1 (transduced microglia). Note the presence of numerous GFP-positive and Iba1-negative cells in all three regions. Scale bars; 50 µm. **(c)** Summary graph showing cell specificity of microglia in the three brain regions as assessed by the percentage of Iba1 (+) cells among GFP (+) cells. GFP; enhanced green fluorescent protein, GL; granule cell layer, Iba1; ionized calcium-binding adaptor molecule 1, ITR; inverted terminal repeat, ML; molecular layer, PGK; phosphoglycerate kinase, polyA; polyadenylation, WPRE; woodchuck hepatitis virus posttranscriptional regulatory element.

Supplementary Figure 2

a

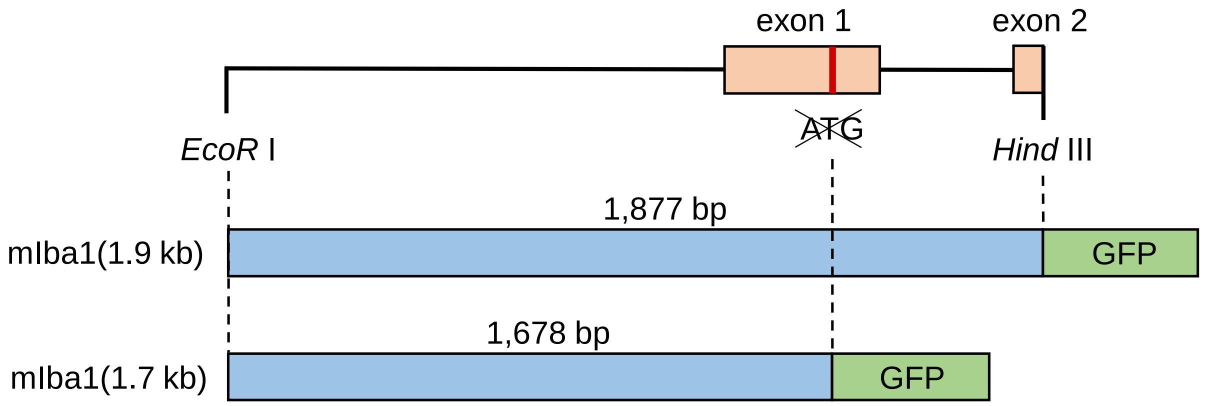

b *Iba1* promoter: 1,678 bp

5’-TACTATAGGATGCATCGTGAAAACCTCGTTTCCACCAGGAACTGAGGTTGCTGCTGGAG
GAAATCTCCCATGAAGAGCTGTTAACTGGGGAACTGGTTGGATCCAGCTCCTACAAGCT
CTCCTTTAATTTCCATACACATGCCAAGCTGTGAACACCTGCACACACAGTACAAATAAA
ACAGTAGAAATGAAATGAAAATTAAAACAAAAACAGCATACTTTCTCCTTTCTACCCACAT
ATCTCCTGCATCCCTGAGAGCAATTTCTTGGCCATCTTCCATAATCATGCTATTCAAACC
TGCCCTCTAAGTACAGAGCTCTGAATGGAGACCACGGGAGCAGACTTTTCATCTGTTGG
CTCCCTGGGCCTAGCAGTGTACCCTGCAAACAGGAAACCTTCGCTCATCATTTATCACA
TGAATGTGGCTAGAGAACTGTCCCCACCAGACACTGAGAGCCTCTCACAGGGCCAGAG
GGTGGCTGCCTCAAAGGCAGGCGAGAGGCTGCAGTCTTCTGCAGAGTGGACAGATACT
GCCTGCCATACAAGGGGACATAGCCTGGGGCGGGGGGCACAAGGGAAGGGGCCTGA
GTGCATTGGAGTCACAGCCCTCCCTCCCCTGGTGACCCAGTGACTGAGGGGACCAAGG
CTATCCCTGGTATGAGGGGAGGGCATTTGCATGAGTTAGACCCCTGACAGCCCATGGT
GGGGACAGGAAGTAGCTCTGTCTACTGTCTCTTTCAGTCTCACTTTCTGTTCCTCCAAA
GTGTCTCTTCAAACTCTCAAATGACTTTGTAGATTCAGCCTAAGATCTCAAAGCAGGTGA
GGCAGGAGGCTGGCACTCAAGAGCCTGACGAACACAAACCCAAGTCCTTCTGGGACAT
GTCTCCATGTGTCCCCAGTGCTCCTCTGTAGCCCCTGAGAGAGGTTTCTTTCCTTTCCA
GGGCGCTAGGCTCAGCTCACCCCATTCCTGGAGCAGCCTGCAGACTTCATCCTCTCTC
TTCCATCCCGGGGAAAGTCAGCCAGTCCTCCTCAGCTGCCTGTCTTAACCTGCATCATG
AAGCCTGAGGAGATTTCAAGTAAACCCTCCCAAGCCCCACCTCAGGATCTGGGGAAAG
CCACTGTCTACCGCATCCTTGGTTTGAGACAGGTTCTTACTGTGTTGGCCTGGCTGGGT
TCCAACTCCCAGGATCCTCTAGCCTCAGCCTCTCTTAGGTGCTAGATTGCAGGCCTAGG
GTATCACGCTCGGTCCCTCAGATCCTGGTGCCTTTCCACACCTCCGGGAGCTGATCTAA
GTCTTTCTCCCACGTACAGTCTGCTTCTTTGGAGGAGCTGTGTGGAGCTAGGTGTGTTA
CAGGGCTGTAGCTCCAGCCATCTGGGAGGCTGAGGCAGGATGGCTACTTCAATTCTCA
AGAGCCTGGATAGGAGACCCTGCCCCCACCCCACCCCCAAAGTAATTTTTCAGCACATT
ACTTCTTCATCTCCTCTCTCAACCCGTTCTGCCTTCTCCTGGGGTGCTGGTGTCAGCAG
AAGCTGATGTGGAAGTGATGCCTGGGAGTTAGCAAGGGAATGAGTGGAAAGGGGAAGT
GTGAGAACGGTCCCAGAAGAGACTGGGGAGCTGGTGGAGAGAGGACCCAGCGGACAG
ACTGCCAGCCTAAGACAACCAGCGTCTGAGGAGCC -3’

Supplementary figure 2. Relative position and sequence of mouse Iba1 promoter. (a) Schema depicting mouse *Iba1* promoter used for generation of Iba1-GFP transgenic mice [mIba1 (1.9 kb)] and relative position of mouse *Iba1* promoter incorporated in AAV [mIba1 (1.7 kb)]. **(b)** The sequence of mouse *Iba1* promoter [mIba1 (1.7 kb)] was used in this study.

Supplementary Figure 3

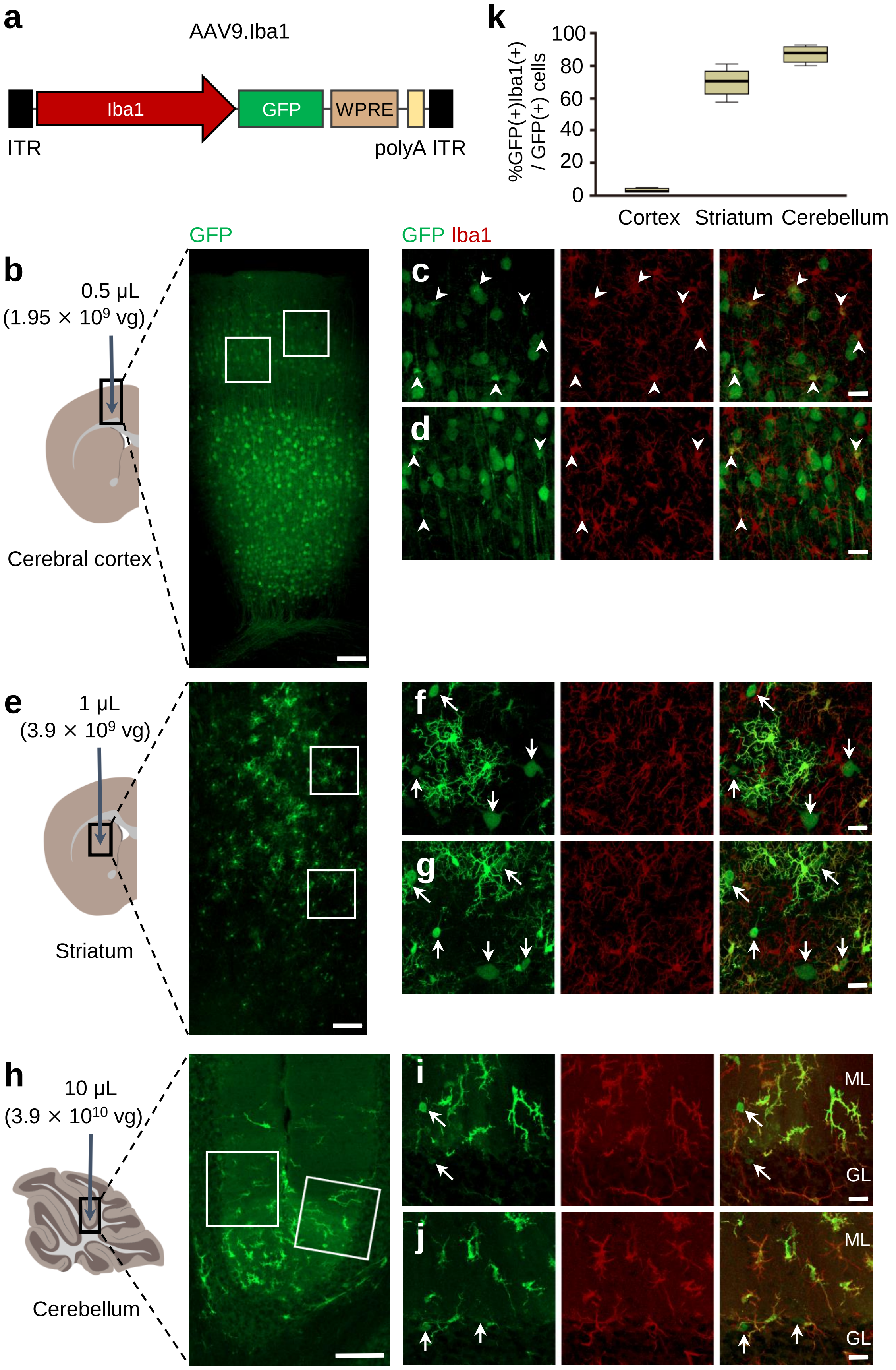

Supplementary figure 3. Microglial-selective transduction in the striatum and cerebellum, but not the cerebral cortex, by AAV9.Iba1. The brain sections were immunostained for GFP and Iba1 one week after the AAV injection. **(a)** Schema of AAV genome comprising the *Iba1* promoter and GFP. **(b-d)** Transduction of numerous Iba1-negative non-microglial cells in the cerebral cortex with minor transduction of microglia (arrowheads). **(e-j)** Robust and efficient transduction of microglia with weak and sparse transduction of Iba1-negative non-microglial cells (arrows) in the striatum **(e–g)** and cerebellum **(h–j)**. Square regions in **(b, e, h)** were enlarged. Scale bars; 100 μm (**b, e, h**) and 20 μm (**c, d, f, g, i, j**). (**k**) Summary graph showing cell specificity of microglia in the three brain regions as assessed by the percentage of Iba1 (+) cells among GFP (+) cells.

Supplementary Figure 4

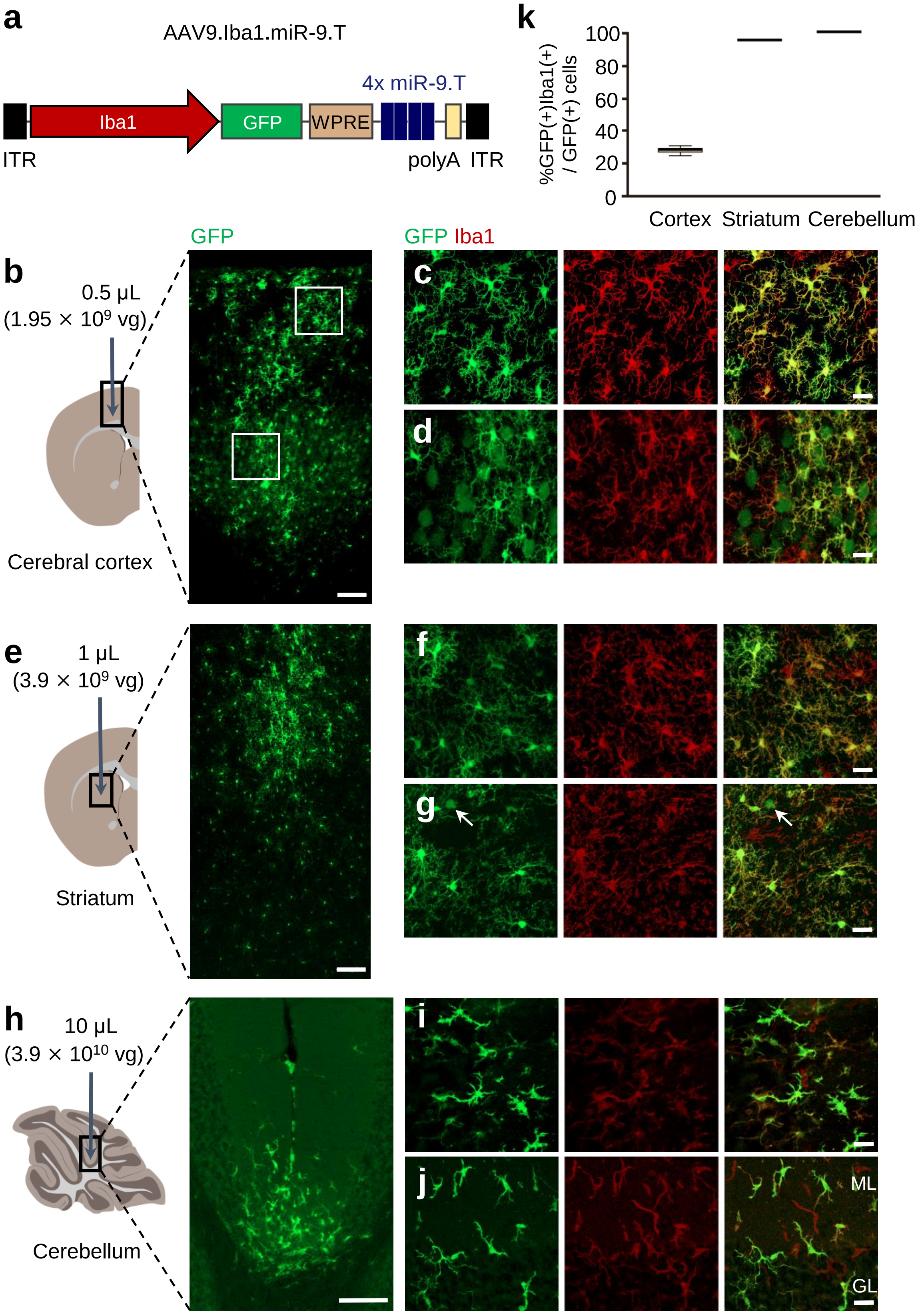

Supplementary figure 4. Addition of miR-9.T to AAV9.Iba1 strengthened detargeting non-microglial cells in the cerebral cortex. Mouse brains were immunolabeled for GFP and Iba1 one week after the AAV vector injection. **(a)** Schema of the AAV construct that expresses GFP and 4 × miR-9.T by the *Iba1* promoter. **(b–d)** Immunohistochemistry of the cerebral cortex. Square regions in **(b)** were enlarged. Note the strong GFP expression in microglia, in contrast to much weaker immunolabeling of non-microglial cells. **(e–j)** Robust and efficient transduction of microglia with rare transduction of Iba1-negative non-microglial cells (arrows) in the striatum **(e–g)** and cerebellum **(h–j)**. Scale bars; 100 μm **(b, e, h)** and 20 μm **(c, d, f, g, i, j)**. **(k)** Summary graph showing cell specificity of microglia in the three brain regions as assessed by the percentage of Iba1 (+) cells among GFP (+) cells.

Supplementary Figure 5

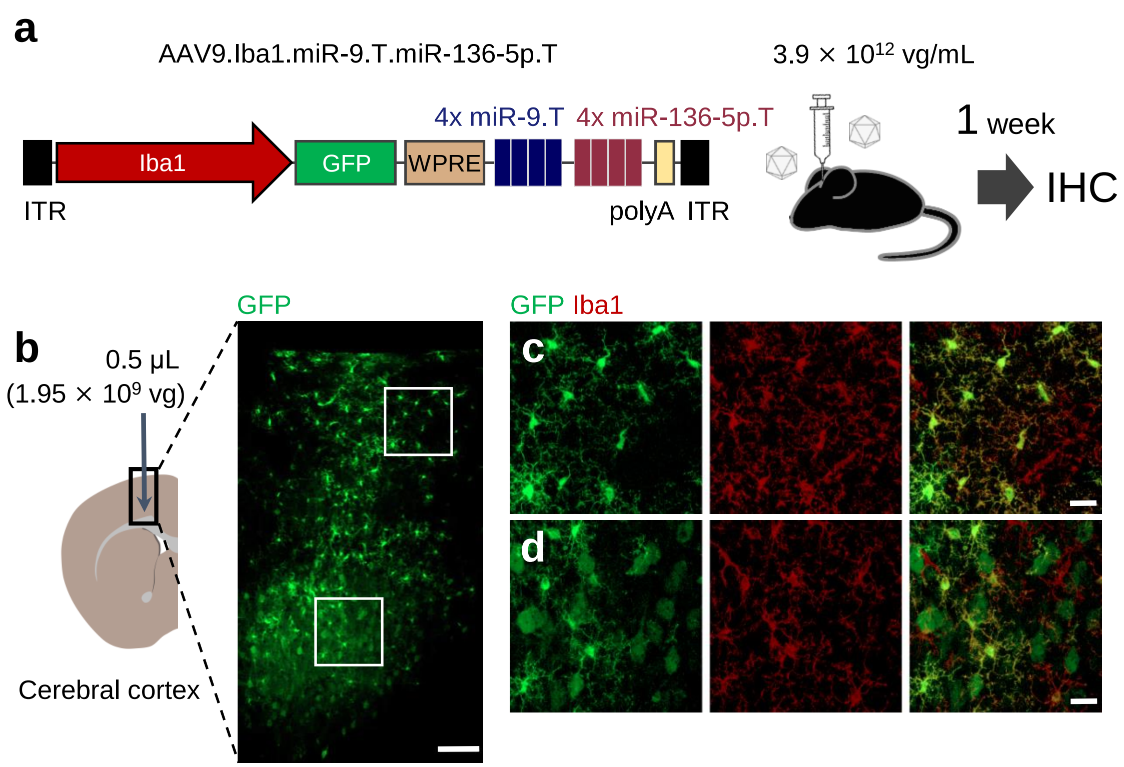

Supplementary figure 5. Failure of further detargeting of cortical neurons by the addition of miR-136-5p.T to AAV9.Iba1.miR-9.T. To suppress insufficient neuronal detargeting in the cerebral cortex by AAV9.Iba1.miR-9.T, quadruplex sequences complementary to miR-136-5p, which is specifically enriched in neurons and depleted in microglia, was inserted into AAV9.Iba1.miR-9.T, following miR-9.T. One week after the viral injection into the cerebral cortex, the cortex was immunostained for GFP and Iba1. **(a)** Schema of AAV genome. **(b)** Diagram depicting the virus injection site. Low-magnified image of the cerebral cortex showing efficient transduction of cortical cells. Scale bar; 100 μm. **(c, d)** Enlarged images of the square regions in **(b)**, showed weak, but obvious transduction of numerous Iba1-negative cells (presumably neurons) in the deeper area of the cerebral cortex **(d)**. Scale bar; 20 μm.

Supplementary Figure 6

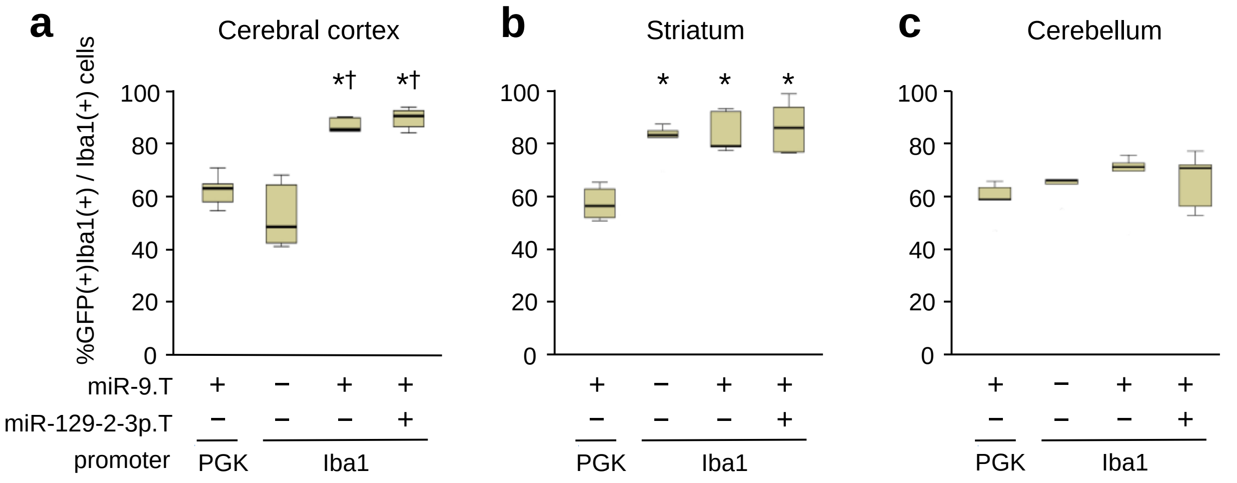

Supplementary figure 6. Quantitative analysis of microglial transduction efficiency by AAV vectors in the cerebral cortex, striatum and cerebellum one week after injection. AAV vectors were injected as shown in Fig. 1. The transduction efficiency of microglia was assessed by the percentage of GFP- and Iba1-double-positive cells (transduced microglia) divided by total count of microglia immunoreactive for Iba1. **(a)** Transduction efficiency in the cerebral cortex (n = 5 mice per group, one-way ANOVA: $F_{(3,16)} = 26.167$, $P < 0.001$; Bonferroni post hoc analysis: $^*P \leq 0.001$ for vs. PGK.miR-9.T, $^{\dagger}P \leq 0.001$ for vs. Iba1). AAV9.PGK.miR-9.T transduced 61.9 ± 5.2% (530 double-positive cells in 855 Iba1-positive cells, n = 5 mice), while AAV9.Iba1.miR-9.T transduced 85.0 ± 6.3% (789 double-positive cells in 927 Iba1-positive cells, n = 5 mice). **(b)** Transduction efficiency in the striatum (n = 5 mice per group, one-way ANOVA: $F_{(3,16)} = 14.562$, $P < 0.001$; Bonferroni post hoc analysis: $^*P \leq 0.001$ for vs. PGK.miR-9.T). AAV9.PGK.miR-9.T transduced 57.1 ± 6.5% (485 double-positive cells in 855 Iba1-positive cells, n = 5 mice), while AAV9.Iba1.miR-9.T transduced 83.9 ± 7.9% (661 double-positive cells in 793 Iba1-positive cells, n = 5 mice). **(c)** Transduction efficiency in the cerebellum (n = 5 mice per group, one-way ANOVA: $F_{(3,16)} = 0.736$, $P = 0.546$). AAV9.PGK.miR-9.T transduced 59.3 ± 7.5% (495 double-positive cells in 836 Iba1-positive cells, n = 5 mice), while AAV9.Iba1.miR-9.T transduced 67.5 ± 12.6% (574 double-positive cells in 853 Iba1-positive cells, n = 5 mice). Note that in the presence of miR-9.T, replacement of the PGK promoter by the *Iba1* promoter significantly increased the transduction efficiency in the cerebral cortex and striatum, while the addition of the miR-129-2-3p.T to AAV9.Iba1.miR-9.T did not affect transduction efficiency in the three brain regions examined.

Supplementary Figure 7

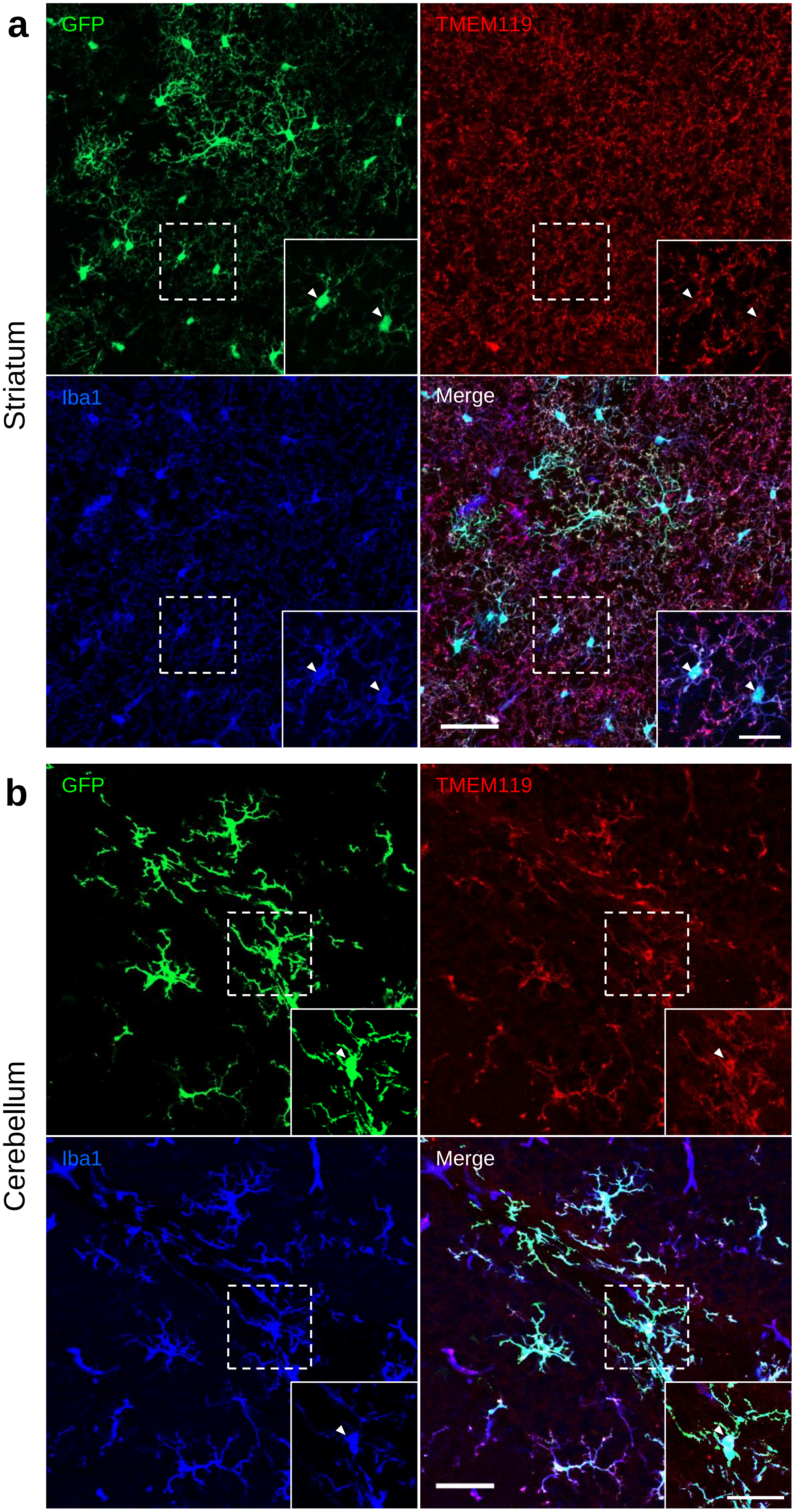

Supplementary figure 7. Absence of GFP-labeled infiltrating monocytes in striatal and cerebellar tissues treated with AAV9.Iba1.miR-9.T.miR-129-2-3p.T. Mice received striatal or cerebellar parenchymal injection of AAV9.Iba1.miR-9.T.miR-129-2-3p.T as depicted in Fig. 2. One week after the injection, striatal and cerebellar sections were triple immunolabeled for GFP, Iba1 and transmembrane protein 119 (TMEM119), a highly specific microglial marker that is not expressed by CNS macrophages, dendritic cells, infiltrating monocytes or other immune or neural cell types. **(a)** Striatal sections. **(b)** Cerebellar sections. Scale bars, 50 μm and 20 μm (inset).

Supplementary Figure 8

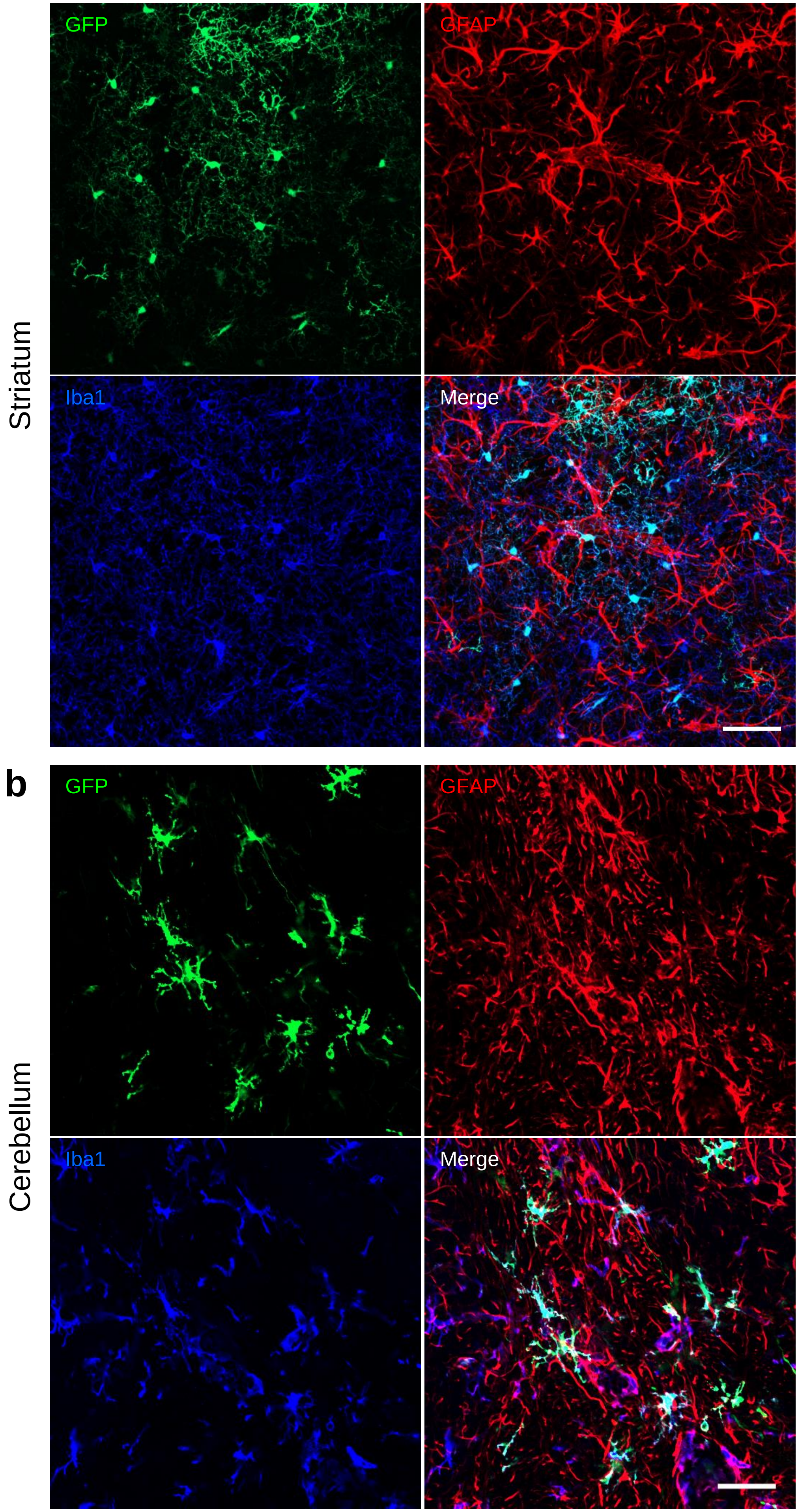

Supplementary figure 8. Absence of astrocyte transduction in striatal and cerebellar tissues treated with AAV9.Iba1.miR-9.T.miR-129-2-3p.T. Mice were treated as depicted in Supplementary figure 7. Striatal and cerebellar sections were triple immunolabeled for GFP, Iba1 and glial fibrillary acidic protein (GFAP), a marker for astrocyte. **(a)** Striatal sections. **(b)** Cerebellar sections. Scale bars, 50 μm.

Supplementary Figure 9

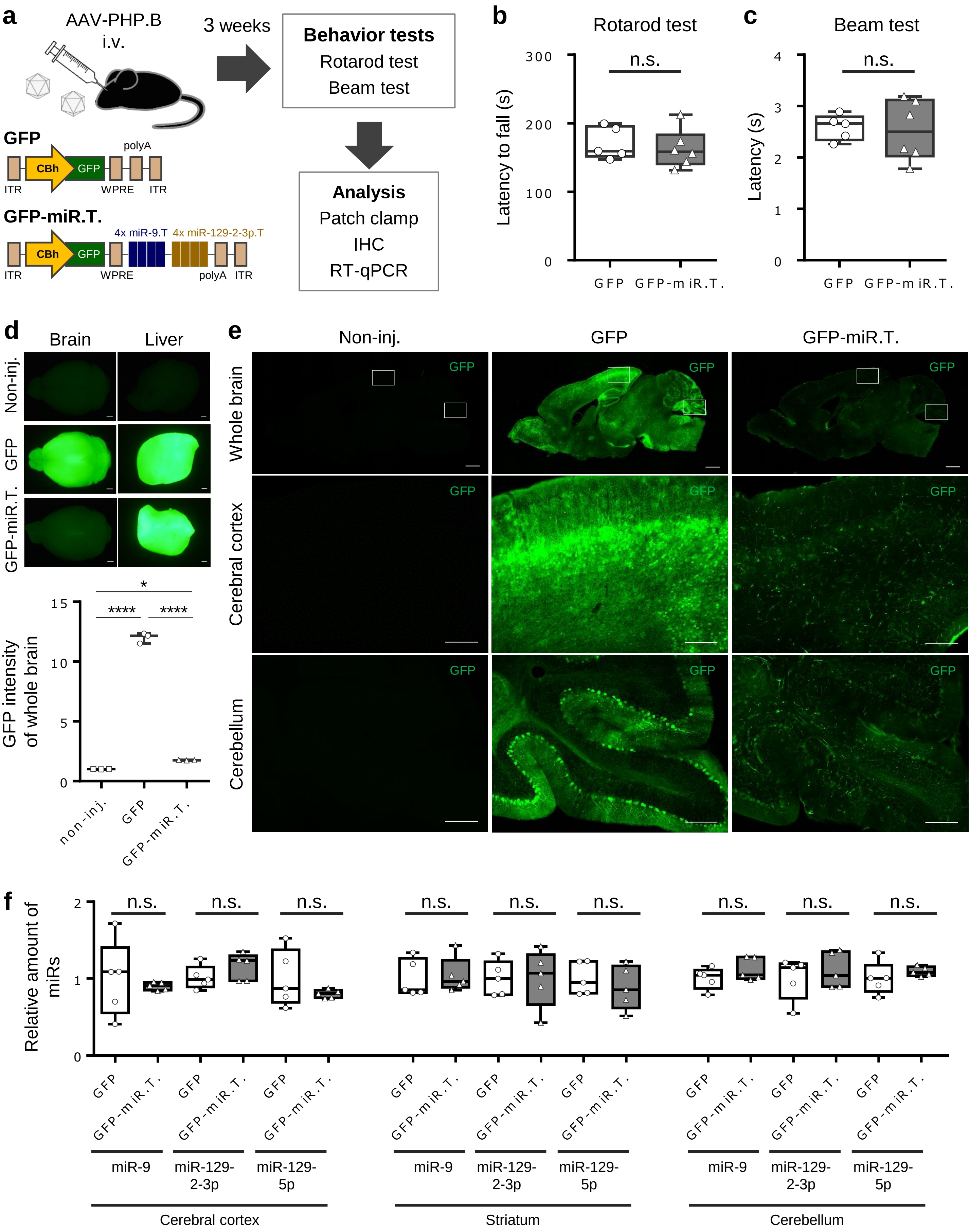

Supplementary figure 9. No influence on motor performance and microRNA levels in three brain regions by overexpression of miR-9.T and miR-129-2-3p.T throughout the brain. (a) Schema depicting an experimental procedure. Mice received intravenous infusion of blood-brain barrier-penetrating AAV-PHP.B expressing GFP-miR-9.T-miR-129-2-3p.T (GFP-miR.T) or GFP alone by the strong CBh promoter. Three weeks after the injection, mice were subjected to behavioral tests, followed by patch-clamp, IHC, and RT-qPCR. **(b, c)** Results of rotarod **(b)** and beam-walking **(c)** tests. There were no significant differences between GFP and GFP-miR.T groups (rotatod test, n = 5 mice per group, two-tailed unpaired *t*-test: $t_{(8)} = 0.489$, $P = 0.585$; beam-walking test, n = 5 mice per group, two-tailed unpaired *t*-test: $t_{(8)} = 0.198$, $P = 0.848$). **(d)** Native GFP fluorescence images of the whole brain and liver, showing GFP expression in the liver, but not in the brain from mice expressing GFP-miR-9.T- miR-129-2-3p.T (bottom panels), in contrast to strong GFP expression in both liver and brain from mice expressing GFP alone (middle panels). The lower graph shows GFP fluorescence intensity (n = 3 mice per group, one-way ANOVA: $F_{(2,6)} = 1789.5$, $P < 0.001$; Tukey post hoc analysis: $*P < 0.05$, $****P < 0.0001$). **(e)** Immunohistochemistry of sagittal sections of the brains presented in **(d)**, which were double immunolabeled for GFP and NeuN, confirming marked suppression of GFP expression in the brain expressing GFP-miR-9.T- miR-129-2-3p.T (only the green channel was presented). Scale bars, 1 mm for whole brains and 200 μm for magnified sections. **(f)** Levels of microRNAs (miR-9, miR-129-2-3p, and miR-129-2-5p) in the cerebral cortex, striatum and cerebellum by RT-qPCR. All pairs of GFP versus GFP-miR.T medians in 3 brain regions were found to be no different (**cerebral cortex**, n = 5 mice per group, two-tailed unpaired *t*-test: $t_{(8)} = 0.448$, $P = 0.667$ for miR-9, $t_{(8)} = 1.342$, $P = 0.217$ for miR-129-2-3p, $t_{(8)} =1.202$, $P = 0.264$ for miR-129-5p; **striatum**, n = 5 per group, two-tailed unpaired *t*-test: $t_{(8)} = 0.263$, $P = 0.799$ for miR-9, $t_{(8)} = 0.004$, $P = 0.997$ for miR-129-2-3p, $t_{(8)} = 0.749$, $P = 0.475$ for miR-129-5p; **cerebellum**, n = 5 mice per group, two-tailed unpaired *t*-test: $t_{(8)} = 1.301$, $P = 0.229$ for miR-9, $t_{(8)} = 0.640$, $P = 0.540$ for miR-129-2-3p, $t_{(8)} = 0.905$, $P = 0.392$ for miR-129-5p). CBh; cytomegalovirus and chicken β-actin hybrid promoter, n.s.; not significant, RT-qPCR; reverse transcription-quantitative polymerase chain reaction.

Supplementary Figure 10

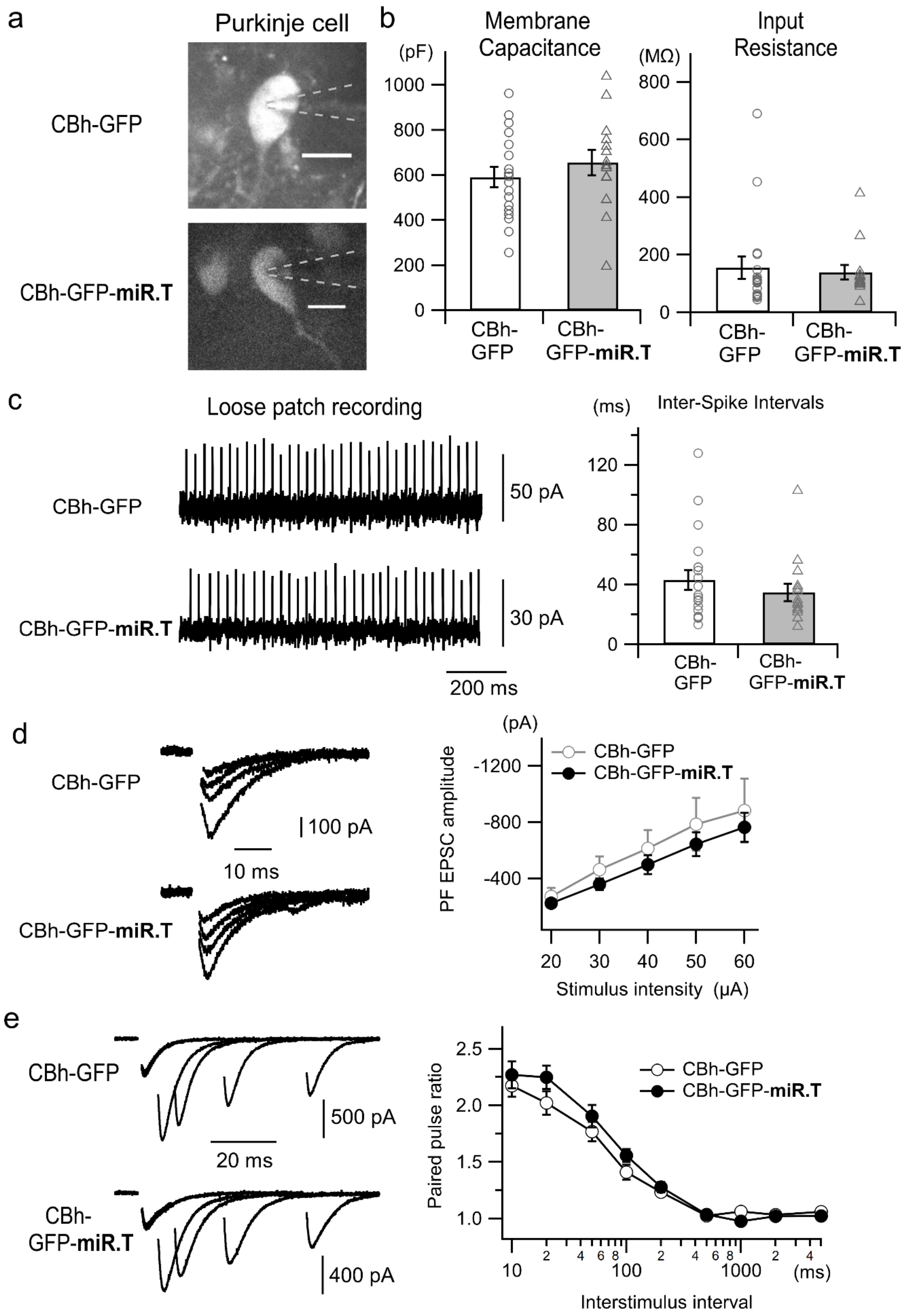

Supplementary figure 10. No effect of miR-9.T and miR-129-2-3p.T overexpression on neuronal functions of cerebellar Purkinje cells. The experimental procedure is shown in supplementary Fig. 8a. **(a)** Representative GFP images of PC somata (i.e. recording sites) transduced with GFP or GFP-miR-9.T-miR-129-2-3p.T (GFP-miR.T) genes. Scale bars, 20 μm. Broken lines delineate recording patch electrodes. Note much weaker GFP signals in a PC transduced with GFP-miR.T (lower panel) than with GFP alone (upper panel), although GFP signals were still detectable in the case of CBh-GFP-miR.T overexpression AAV vector. **(b)** Passive electrical membrane properties of PCs transduced with GFP or GFP-miR.T. No difference in membrane capacitance (left panel) or input resistance (right panel) of the recorded PCs between CBh-GFP and CBh-GFP-miR.T. Symbols and bar graphs indicate individual data points and mean ± SEM, respectively in this figure. **(c)** The left panel shows representative traces of spontaneous spike firing in PCs recorded near the PC somata with the extracellular loose cell-attached patch-clamp technique. The right panel shows pooled data of inter-spike intervals, which were measured as the averaged values of all the intervals between detected spikes during the recording time (60 s) in single PCs. Spontaneous firing properties were normal in PCs expressing miR.T exogenously. **(d)** Left panel, representative overlaid traces of PF EPSCs evoked with various stimulus intensities in PCs expressing GFP or GFP-miR.T. Right graph, the relationship between electrical stimulus intensity and PF EPSC amplitude (mean ± SEM). **(e)** Left panel, overlaid traces of PF EPSCs evoked by paired-pulse stimulation protocols with four different intervals. Right graph, paired-pulse ratios (the second EPSC amplitudes normalized to the first ones) were plotted against varied interstimulus intervals of the two pulses. Basal synaptic properties and short-term synaptic plasticity at PF-PC synapses were normal in PCs overexpressing miR-T sequences. EPSC; excitatory postsynaptic current, PC; Purkinje cell, PF; parallel fiber, SEM; standard error of the mean.

Supplementary Figure 11

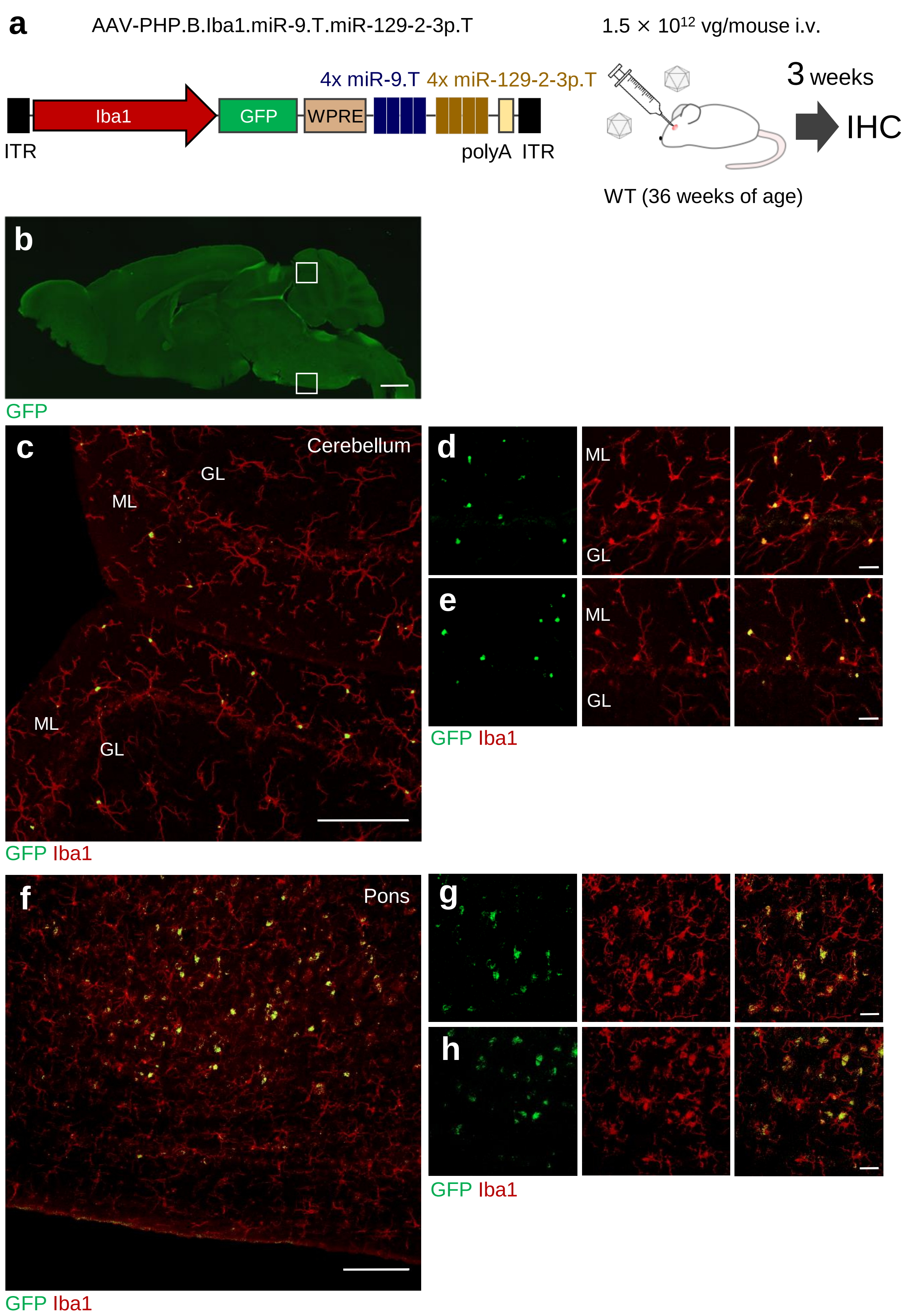

Supplementary figure 11. Successful microglia targeting, but aggregate formation in wild-type mice by intravenous administration of AAV-PHP.B. (a) Diagram showing the AAV construct and experimental procedure. Wild-type mice at 36 weeks of age received intravenous infusion of AAV-PHP.B.Iba1.miR-9.T.miR-129-2-3p.T (dose: 1.5 x 10^{12} vg/mouse). Three weeks after the viral injection, brain sections were produced and analyzed by double immunolabeling for GFP and Iba1. **(b)** Sagittal brain section of the brain, in which square regions were magnified **(c, f)**. **(c-e)** Immunofluorescent images of a cerebellar section. **(f-h)** Immunofluorescent images of a pontine nucleus. Note aggregation of GFP in microglia. Scale bars, 1 mm **(b)** and 20 μm.

Supplementary Figure 12

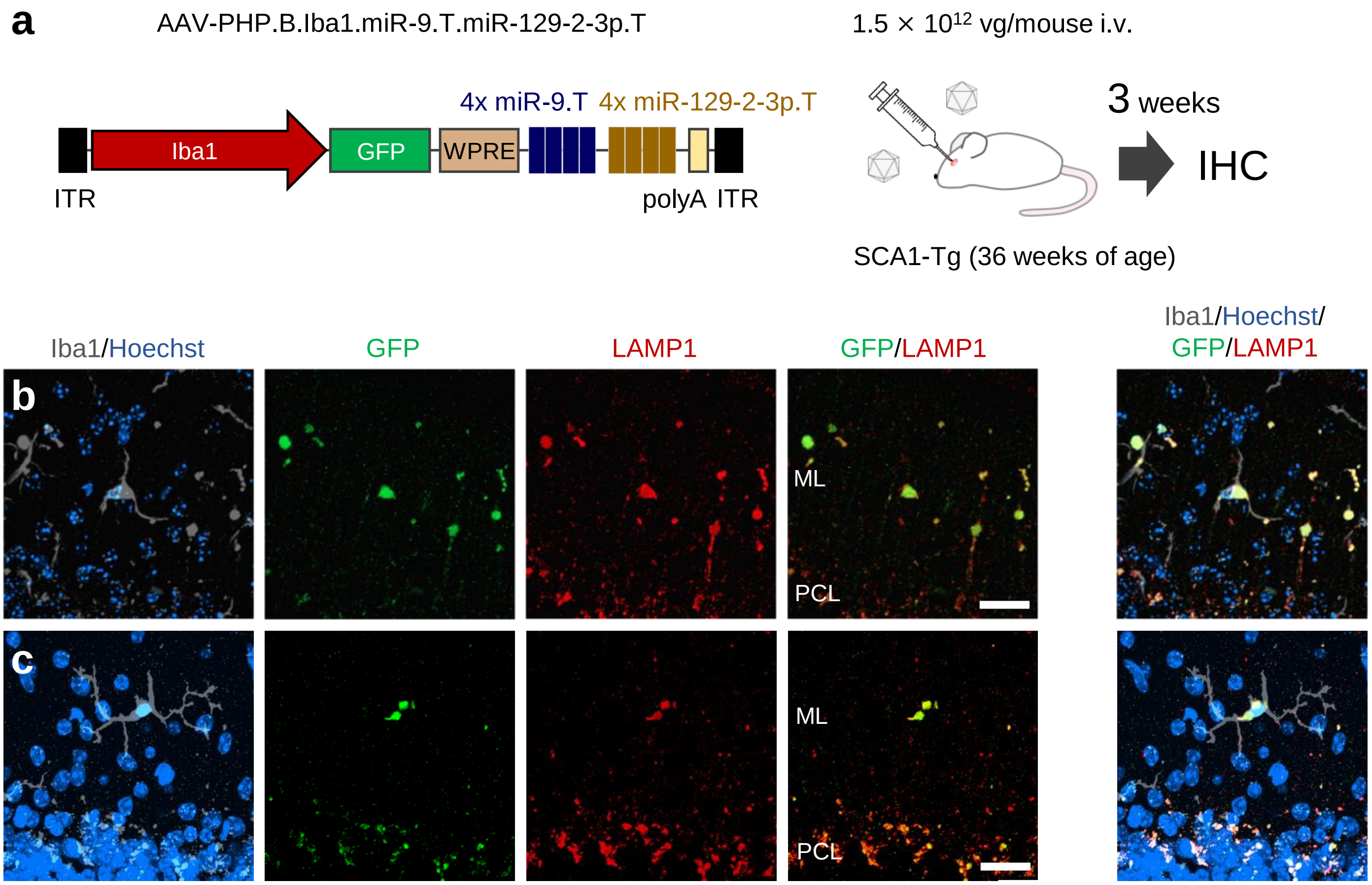

Supplementary figure 12. Localization of GFP aggregates in microglial lysosomes. (a) Diagram showing the AAV construct and experimental procedure. SCA1-Tg mice at 36 weeks of age received intravenous infusion of AAV-PHP.B.Iba1.miR-9.T.miR-129-2-3p.T (dose: 1.5 x 10^{12} vg/mouse). Three weeks after the viral injection, brain sections were produced and analyzed by immunolabelling for Iba1, nuclear DNA (by Hoechst stain), GFP, and LAMP1, a glycoprotein located across lysosomal membranes. **(b-c)** Immunofluorescent images of a sagittal section of the cerebellum. Note that GFP aggregates, which are located in the cytoplasm of microglia, precisely match LAMP1 immunoreactivity, indicating localization of GFP in the lysosomes. Scale bars, 20 µm. LAMP1; lysosomal-associated membrane protein 1, PCL; Purkinje cell layer, SCA1-Tg; spinocerebellar ataxia type 1-transgenic.

Supplementary Table 1

miRT		Oligo sequence (5' → 3')
miR9T	S1	CTAATCATACAGCTAGATAACCAAAGCGATTCATACAGCTAGATAACCAAAGACGCGT
	S2	TCATACAGCTAGATAACCAAAGTCACTCATACAGCTAGATAACCAAAGG
	AS1	CTTTGGTTATCTAGCTGTATGAATCGCTTTGGTTATCTAGCTGTATGATTAGgtac
	AS2	gatcCCTTTGGTTATCTAGCTGTATGAGTGACTTTGGTTATCTAGCTGTATGAACGCGT
miR9T-miR129-2-3pT	S1	CTCATACAGCTAGATAACCAAAGCGATTCATACAGCTAGATAACCAAAGCaattG
	S2	TCATACAGCTAGATAACCAAAGTCACTCATACAGCTAGATAACCAAAGGCTAGC
	S3	ATGCTTTTTGGGGTAAGGGCTTCGATATGCTTTTTGGGGTAAGGGCTTCttaaG
	S4	ATGCTTTTTGGGGTAAGGGCTTTCACATGCTTTTTGGGGTAAGGGCTTG
	AS1	CTTTGGTTATCTAGCTGTATGAATCGCTTTGGTTATCTAGCTGTATGAGgtac
	AS2	CTTTGGTTATCTAGCTGTATGAGTGACTTTGGTTATCTAGCTGTATGACaattG
	AS3	AAGCCCTTACCCCAAAAAGCATATCGAAGCCCTTACCCCAAAAAGCATGCTAGC
	AS4	gatcCAAGCCCTTACCCCAAAAAGCATGTGAAAGCCCTTACCCCAAAAAGCATCttaaG
miR9T-miR136-5pT	S1	CTCATACAGCTAGATAACCAAAGCGATTCATACAGCTAGATAACCAAAGCaattG
	S2	TCATACAGCTAGATAACCAAAGTCACTCATACAGCTAGATAACCAAAGGCTAGC
	S3	CCATCATCAAAACAAATGGAGTCGATCCATCATCAAAACAAATGGAGTCttaaG
	S4	CCATCATCAAAACAAATGGAGTTCACCCATCATCAAAACAAATGGAGTG
	AS1	CTTTGGTTATCTAGCTGTATGAATCGCTTTGGTTATCTAGCTGTATGAGgtac
	AS2	CTTTGGTTATCTAGCTGTATGAGTGACTTTGGTTATCTAGCTGTATGACaattG
	AS3	ACTCCATTTGTTTTGATGATGGATCGACTCCATTTGTTTTGATGATGGGCTAGC
	AS4	gatcCACTCCATTTGTTTTGATGATGGGTGAACTCCATTTGTTTTGATGATGGCttaaG

Supplementary Table 2

Antibodies used in this study

Antibody	Primary/ Secondary	Dilution factor	Source	Identifier
rat anti-GFP	Primary	1,000	Nacalai Tesque	04404-84; RRID:AB_10013361
mouse anti-GFP	Primary	1,000	GeneTex	GTX21218; RRID:AB_371417
rabbit anti-Iba1	Primary	500	FUJIFILM Wako	019-19741; RRID:AB_839504
rat anti-Lamp1 (CD107a)	Primary	500	BD Pharmingen	553792; RRID:AB_2134499
mouse anti-TMEM119	Primary	100 or 500	Synaptic Systems	400 011; RRID:AB_2782984
mouse anti-GFAP	Primary	400	Sigma-Aldrich	G3893; RRID:AB_477010
mouse anti-NeuN	Primary	1000	Sigma-Aldrich	MAB377; RRID:AB_2298772
Alexa Fluor 488 donkey anti-rat IgG	Secondary	1,000	Thermo Fisher	A21208; RRID:AB_141709
Alexa Fluor 488 donkey anti-mouse IgG	Secondary	1,000	Thermo Fisher	A21202; RRID:AB_141607
Alexa Fluor 594 donkey anti-rabbit IgG	Secondary	1,000	Thermo Fisher	A21207; RRID:AB_141637
Alexa Fluor 594 donkey anti-rat IgG	Secondary	1,000	Thermo Fisher	A21209; RRID:AB_2535795
Alexa Fluor 680 donkey anti-rabbit IgG	Secondary	1,000	Thermo Fisher	A10043; RRID:AB_2534018
Alexa Fluor Plus 488 donkey anti-rat IgG	Secondary	2,000	Thermo Fisher	A48269; RRID:AB_2893137
Alexa Fluor Plus 555 donkey anti-mouse IgG	Secondary	2,000	Thermo Fisher	A32773; RRID:AB_2762848
Alexa Fluor Plus 555 donkey anti-rabbit IgG	Secondary	2,000	Thermo Fisher	A32794; RRID:AB_2762834
Alexa Fluor Plus 647 donkey anti-rabbit IgG	Secondary	2,000	Thermo Fisher	A32795; RRID:AB_2762835
Alexa Fluor Plus 647 donkey anti-mouse IgG	Secondary	2,000	Thermo Fisher	A32787; RRID:AB_2762830

Supplementary Video legends

Supplementary Video 1.

A video of GFP images shows the basal motility of microglial processes depicted in Fig. 6a upper panel. The play speed is 40 times faster than that in real-time.

Supplementary Video 2.

The video of GFP images shows the basal motility of microglial processes depicted in Fig. 6a (lower panel) as a larger field of view.

The cyan rectangle in the video corresponds to the area in Fig. 6a (lower panel). The play speed is 40 times faster than that in real-time.

Supplementary Video 3.

Another example is a GFP video shows basal motility of microglial processes. The play speed is 40 times faster than that in real time.

Supplementary Video 4.

A video of GFP fluorescence shows ATP-induced upregulation of microglial motility depicted in Fig. 6b (upper panel) as a larger field of view. The play speed is 100 times faster than that in real-time.

Supplementary Video 5.

A video of GFP fluorescence shows ATP-induced upregulation of microglial motility depicted in Fig. 6b (lower panel) as a larger field of view. The play speed is 60 times faster than that in real-time.

Supplementary Video 6.

An example of process motility (the same process as in Fig. 7d) was observed by confocal live imaging in the granule cell layer of acute sagittal cerebellar slices expressing G-CaMP7.09, using our AAV-mediated microglia-selective gene delivery method. Images were acquired every 2s. The video plays at 30 frames/s (60 times faster than the real-time).

Supplementary Video 7.

An example of an ATP-induced Ca^{2+} responses and putative microglial process movement in an experimental condition similar to those shown in Supplementary Video 6. Exogenous ATP (100 μM) were bath-applied for approximately 30 s, as indicated in the video. In a pseudo-color scale in the video, “cold-to-hot” colors correspond to “low-to-high” fluorescence values. Images were acquired every 2 s. The video plays at 30 frames/s (60 times faster than the real-time).

Supplementary Video 8.

Another example of an ATP-induced Ca^{2+} responses and microglial motility in an experimental condition similar to Supplementary Video 7. Process motility became visible with some delay after the first ATP application, and was greatly enhanced after the second ATP application in this video. The video plays at 30 frames/s (60 times faster than the real-time).
