# Supplementary material for: Development of microglia-targeting adeno-associated viral vectors as tools to study microglial behavior in vivo
Source: Commun Biol. 2022 Nov 11;5:1224. doi: 10.1038/s42003-022-04200-3 (PMC9652230; doi:10.1038/s42003-022-04200-3)
Supplement: Supplementary file 3 — Description of Additional Supplementary Files [file 42003_2022_4200_MOESM3_ESM.pdf]

Description of Additional Supplementary Files

File name: Supplementary Data 1

Description: Source data.

File name: Supplementary Video 1

Description: This video provides supplementary information to Figure 6a.

File name: Supplementary Video 2

Description: This video provides supplementary information to Figure 6a.

File name: Supplementary Video 3

Description: This video provides supplementary information to Figure 6a.

File name: Supplementary Video 4

Description: This video provides supplementary information to Figure 6b.

File name: Supplementary Video 5

Description: This video provides supplementary information to Figure 6b.

File name: Supplementary Video 6

Description: This video provides supplementary information to Figure 7d.

File name: Supplementary Video 7

Description: This video provides supplementary information to Figure 7d.

File name: Supplementary Video 8

Description: This video provides supplementary information to Figure 7d.
