# Supplementary material for: Development of microglia-targeting adeno-associated viral vectors as tools to study microglial behavior in vivo
Source: Commun Biol. 2022 Nov 11;5:1224. doi: 10.1038/s42003-022-04200-3 (PMC9652230; doi:10.1038/s42003-022-04200-3)
Supplement: Supplementary file 13 — Reporting Summary [file 42003_2022_4200_MOESM13_ESM.pdf]

nature research

Corresponding author(s): DBPR, COMMSBIO-22-2203-T

Last updated by author(s): Jul 13, 2022

Reporting Summary

Nature Research wishes to improve the reproducibility of the work that we publish. This form provides structure for consistency and transparency in reporting. For further information on Nature Research policies, see Authors & Referees and the Editorial Policy Checklist.

Statistics

For all statistical analyses, confirm that the following items are present in the figure legend, table legend, main text, or Methods section.

n/a	Confirmed	
☐	☒	The exact sample size (*n*) for each experimental group/condition, given as a discrete number and unit of measurement
☐	☒	A statement on whether measurements were taken from distinct samples or whether the same sample was measured repeatedly
☐	☒	The statistical test(s) used AND whether they are one- or two-sided<br>*Only common tests should be described solely by name; describe more complex techniques in the Methods section.*
☒	☐	A description of all covariates tested
☐	☒	A description of any assumptions or corrections, such as tests of normality and adjustment for multiple comparisons
☐	☒	A full description of the statistical parameters including central tendency (e.g. means) or other basic estimates (e.g. regression coefficient) AND variation (e.g. standard deviation) or associated estimates of uncertainty (e.g. confidence intervals)
☐	☒	For null hypothesis testing, the test statistic (e.g. F, t, r) with confidence intervals, effect sizes, degrees of freedom and P value noted<br>*Give P values as exact values whenever suitable.*
☒	☐	For Bayesian analysis, information on the choice of priors and Markov chain Monte Carlo settings
☒	☐	For hierarchical and complex designs, identification of the appropriate level for tests and full reporting of outcomes
☒	☐	Estimates of effect sizes (e.g. Cohen's *d*, Pearson's *r*), indicating how they were calculated

Our web collection on statistics for biologists contains articles on many of the points above.

Software and code

Policy information about availability of computer code

Data collection	ZEN blue (Zeiss) for confocal images collection<br>Andor iQ2 (Andor) for acquisition of control calcium imaging data
Data analysis	Andor iQ2 (Andor), ImageJ 1.52p, and Igor Pro8 (WaveMetrics) for calcium image processing and analysis<br>image stabilizer plugin for ImageJ provided by K. Li (http://www.cs.cmu.edu/~kangli/code/Image_Stabilizer.html) for correction of calcium image drift<br>IBM SPSS Statistics 22 (IBM) for statistical analysis

For manuscripts utilizing custom algorithms or software that are central to the research but not yet described in published literature, software must be made available to editors/reviewers. We strongly encourage code deposition in a community repository (e.g. GitHub). See the Nature Research guidelines for submitting code & software for further information.

Data

Policy information about availability of data

All manuscripts must include a data availability statement. This statement should provide the following information, where applicable:

- Accession codes, unique identifiers, or web links for publicly available datasets
- A list of figures that have associated raw data
- A description of any restrictions on data availability

The data that support the findings of this study are available from the corresponding author upon reasonable request.

Field-specific reporting

Please select the one below that is the best fit for your research. If you are not sure, read the appropriate sections before making your selection.

☒ Life sciences ☐ Behavioural & social sciences ☐ Ecological, evolutionary & environmental sciences

For a reference copy of the document with all sections, see nature.com/documents/nr-reporting-summary-flat.pdf

Life sciences study design

All studies must disclose on these points even when the disclosure is negative.

Sample size	No statistical methods were used to pre-determine sample size but our sample sizes were similar to those reported in previous studies.
Data exclusions	No data were excluded from our studies.
Replication	Number of animals per group are treated as biological replicates. Number of replicates (n) are indicated in the main text and figure legends.
Randomization	The animals were randomly assigned to different experimental groups.
Blinding	Data collection and analysis were not performed blind to the conditions of the experiments.

Reporting for specific materials, systems and methods

We require information from authors about some types of materials, experimental systems and methods used in many studies. Here, indicate whether each material, system or method listed is relevant to your study. If you are not sure if a list item applies to your research, read the appropriate section before selecting a response.

Materials & experimental systems

n/a	Involved in the study	
☐	☒	Antibodies
☒	☐	Eukaryotic cell lines
☒	☐	Palaeontology
☐	☒	Animals and other organisms
☒	☐	Human research participants
☒	☐	Clinical data

Methods

n/a	Involved in the study	
☒	☐	ChIP-seq
☒	☐	Flow cytometry
☒	☐	MRI-based neuroimaging

Antibodies

Antibodies used

rat anti-GFP (id. 04404-84, monoclonal, Nacalai Tesque, 1:1000)
rat anti-Lamp1 (id. 553792, monoclonal, BD Pharmingen, 1:500)
rabbit anti-Iba1 (id. 019-19741, polyclonal, Wako, 1:500)
mouse anti-GFP (id. GTX21218, monoclonal, GeneTex, 1:1000)
mouse anti-TMEM119 (id. 400 011, monoclonal, Synaptic Systems, 1:100 or 1:500)
mouse anti- GFAP (id. G3893, monoclonal, Sigma-Aldrich, 1:400)
mouse anti-NeuN (id. MAB377, monoclonal, Sigma-Aldrich, 1:1000)
donkey anti-rat Alexa Fluor 488 (id. A21208, polyclonal, Thermo Fisher, 1:1000)
donkey anti-rat Alexa Fluor 594 (id. A21209, polyclonal, Thermo Fisher, 1:1000)
donkey anti-rabbit Alexa Fluor 594 (id. A21207, polyclonal, Thermo Fisher, 1:1000)
donkey anti-rabbit Alexa Fluor 680 (id. A10043, polyclonal, Thermo Fisher, 1:1000)
donkey anti-mouse Alexa Fluor 488 (id. A21202, polyclonal, Thermo Fisher, 1:1000)
donkey anti-rat Alexa Fluor Plus 488 (id. A48269, polyclonal, Thermo Fisher, 1:2000)
donkey anti-rabbit Alexa Fluor Plus 647 (id. A32795, polyclonal, Thermo Fisher, 1:2000)
donkey anti-rabbit Alexa Fluor Plus 555 (id. A32794, polyclonal, Thermo Fisher, 1:2000)
donkey anti-mouse Alexa Fluor Plus 555 (id. A32773, polyclonal, Thermo Fisher, 1:2000)
donkey anti-mouse Alexa Fluor Plus 647 (id. A32787, polyclonal, Thermo Fisher, 1:2000)

Validation

rat anti-GFP: http://www.nacalaiusa.com/products/view/101/anti-gfp-rat-igg2a-monoclonal-gf090r
rat anti-Lamp1: https://www.bdbiosciences.com/en-us/products/reagents/flow-cytometry-reagents/research-reagents/single-color-antibodies-ruo/purified-rat-anti-mouse-cd107a.553792
rabbit anti-Iba1: https://labchem-wako.fujifilm.com/us/product/detail/W01W0101-1974.html
mouse anti-GFP: https://www.genetex.com/Product/Detail/GFP-antibody-9F9-F9/GTX21218
mouse anti-TMEM119: https://sysy.com/product/400011
mouse anti-GFAP: https://www.sigmaaldrich.com/JP/en/product/sigma/g3893
mouse anti-NeuN: https://www.merckmillipore.com/JP/ja/product/Anti-NeuN-Antibody-clone-A60,MM_NF-MAB377

Donkey anti-Rat Alexa Fluor 488: https://www.thermofisher.com/antibody/product/Donkey-anti-Rat-IgG-H-L-Highly-Cross-Adsorbed-Secondary-Antibody-Polyclonal/A-21208
Donkey anti-Rat Alexa Fluor 594: https://www.thermofisher.com/antibody/product/Donkey-anti-Rat-IgG-H-L-Highly-Cross-Adsorbed-Secondary-Antibody-Polyclonal/A-21209
Donkey anti-Rabbit Alexa Fluor 594: https://www.thermofisher.com/antibody/product/Donkey-anti-Rabbit-IgG-H-L-Highly-Cross-Adsorbed-Secondary-Antibody-Polyclonal/A-21207
Donkey anti-Rabbit Alexa Fluor 680: https://www.thermofisher.com/antibody/product/Donkey-anti-Rabbit-IgG-H-L-Highly-Cross-Adsorbed-Secondary-Antibody-Polyclonal/A10043
Donkey anti-Mouse Alexa Fluor 488: https://www.thermofisher.com/antibody/product/Donkey-anti-Mouse-IgG-H-L-Highly-Cross-Adsorbed-Secondary-Antibody-Polyclonal/A-21202
Donkey anti-rat Alexa Fluor Plus 488: https://www.thermofisher.com/antibody/product/Donkey-anti-Rat-IgG-H-L-Highly-Cross-Adsorbed-Secondary-Antibody-Polyclonal/A48269
Donkey anti-rabbit Alexa Fluor Plus 555: https://www.thermofisher.com/antibody/product/Donkey-anti-Rabbit-IgG-H-L-Highly-Cross-Adsorbed-Secondary-Antibody-Polyclonal/A32794
Donkey anti-rabbit Alexa Fluor Plus 647: https://www.thermofisher.com/antibody/product/Donkey-anti-Rabbit-IgG-H-L-Highly-Cross-Adsorbed-Secondary-Antibody-Polyclonal/A32795
Donkey anti-mouse Alexa Fluor Plus 555: https://www.thermofisher.com/antibody/product/Donkey-anti-Mouse-IgG-H-L-Highly-Cross-Adsorbed-Secondary-Antibody-Polyclonal/A32773
Donkey anti-mouse Alexa Fluor Plus 647: https://www.thermofisher.com/antibody/product/Donkey-anti-Mouse-IgG-H-L-Highly-Cross-Adsorbed-Secondary-Antibody-Polyclonal/A32787

Animals and other organisms

Policy information about studies involving animals; ARRIVE guidelines recommended for reporting animal research

Laboratory animals	C57BL/6J mice, Male, 4-8 weeks, 24-36 weeks<br>FVB/N-Tg (Pcp2-ATXN1*82Q)5Horr mice, Male, 24-36 weeks
Wild animals	This study did not involve wild animals.
Field-collected samples	This study did not involve samples collected from field.
Ethics oversight	All animal protocols were approved by the Japanese Act on the Welfare and Management of Animals and Guidelines for Proper Conduct of Animal Experiments as issued by the Science Council of Japan.

Note that full information on the approval of the study protocol must also be provided in the manuscript.
